# Supplementary material for: Vitamin D Modified DSS-Induced Colitis in Mice via STING Signaling Pathway
Source: Biology (Basel). 2025 Jun 18;14(6):715. doi: 10.3390/biology14060715 (PMC12190092; doi:10.3390/biology14060715)
Supplement: Supplementary file 1 [file biology-14-00715-s001.zip › Supplementary Materials S1: 16s-report of gut microbiota/Groups/diff_analysis/metagenomeRSpecies/figure/barplot/top10log.pdf]

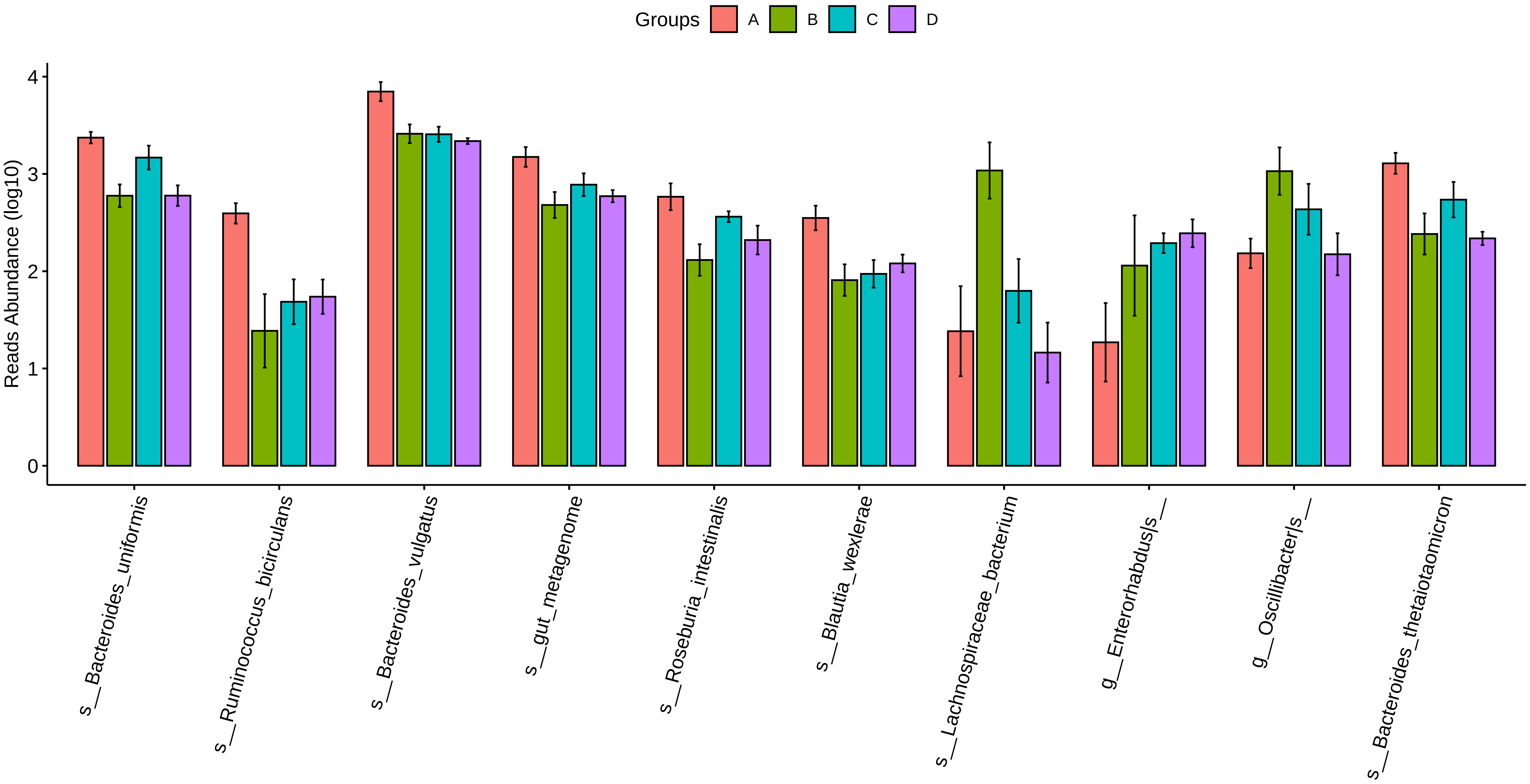
Groups
A
B
C
D
Reads Abundance (log10)
0
1
2
3
4
s__Bacteroides_uniformis
s__Ruminococcus_bicirculans
s__Bacteroides_vulgatus
s__gut_metagenome
s__Roseburia_intestinalis
s__Blautia_wexlerae
s__Lachnospiraceae_bacterium
g__Enterorhabdus|s__
g__Oscillibacter|s__
s__Bacteroides_thetaiotaomicron
